# Multidrug-Resistant Bacteria in Surgical Intensive Care Units: Antibiotic Susceptibility and β-Lactamase Characterization

**DOI:** 10.3390/pathogens13050411

**Published:** 2024-05-15

**Authors:** Daniela Bandić Pavlović, Mladen Pospišil, Marina Nađ, Vilena Vrbanović Mijatović, Josefa Luxner, Gernot Zarfel, Andrea Grisold, Dinko Tonković, Mirela Dobrić, Branka Bedenić

**Affiliations:** 1 Department of Anesthesiology and Intensive Care, University of Zagreb School of Medicine, University Hospital Centre Zagreb, 10000 Zagreb, Croatia; daniela.bandic@mef.hr (D.B.P.); vilena.v@gmail.com (V.V.M.); dinko.tonkovic@mef.hr (D.T.); 2 Department of Emergency Medicine, University Hospital Centre Zagreb, 10000 Zagreb, Croatia; mladenpospisil@gmail.com; 3 University of Zagreb School of Medicine, 10000 Zagreb, Croatia; mnad@student.mef.hr; 4 Institute for Hygiene, Microbiology and Environmental Medicine, Medical University of Graz, 8010 Graz, Austria; josefa.luxner@medunigraz.at (J.L.); gernot.zarfel@medunigraz.at (G.Z.); andrea.grisold@medunigraz.at (A.G.); 5 Department of Anesthesiology, Intensive Medicine and Pain Management, University Hospital Centre Sestre Milosrdnice, 10000 Zagreb, Croatia; dobric.mirela@gmail.com; 6 Biomedical Research Center Šalata—BIMIS, Department for Clinical Microbiology and Infection Prevention and Control, University of Zagreb School of Medicine, University Hospital Centre Zagreb, 10000 Zagreb, Croatia

**Keywords:** *Acinetobacter baumannii*, carbapenemase, Enterobacterales, multidrug-resistant bacteria, surgical intensive care unit

## Abstract

Multidrug-resistant (MDR) bacteria of the utmost importance are extended-spectrum β-lactamase (ESBL) and carbapenemase-producing Enterobacterales (CRE), carbapenem-resistant *Acinetobacter baumannii* (CRAB), carbapenem-resistant *Pseudomonas aeruginosa* (CRPA), methicillin-resistant *Staphylococcus aureus* (MRSA) and vancomycin-resistant *Enterococcus* spp. (VRE). In this study, an evaluation of MDR bacteria in surgical intensive care units in a tertiary referral hospital was conducted. The study aimed to characterize β-lactamases and other resistance traits of Gram-negative bacteria isolated in surgical intensive care units (ICUs). Disk diffusion and the broth dilution method were used for antibiotic susceptibility testing, whereas ESBL screening was performed through a double disk synergy test and an inhibitor-based test with clavulanic acid. A total of 119 MDR bacterial isolates were analysed. ESBL production was observed in half of the *Proteus mirabilis*, 90% of the *Klebsiella pneumoniae* and all of the *Enterobacter cloacae* and *Escherichia coli* isolates. OXA-48 carbapenemase, carried by the L plasmid, was detected in 34 *K. pneumoniae* and one *E. coli* and *Enterobacter cloacae* complex isolates, whereas NDM occurred sporadically and was identified in three *K. pneumoniae* isolates. OXA-48 positive isolates coharboured ESBLs belonging to the CTX-M family in all but one isolate. OXA-23 carbapenemase was confirmed in all *A. baumannii* isolates. The findings of this study provide valuable insight of resistance determinants of Enterobacterales and *A. baumannii* which will enhance surveillance and intervention strategies that are necessary to curb the ever-growing carbapenem resistance rates.

## 1. Introduction

The ever-growing antibiotic resistance rates present a major threat to human health worldwide by limiting therapeutic possibilities, which in turn increases the severity of infection and prolongs hospitalization. Multidrug-resistant bacteria (MDR) are often the causative agents of severe infections in surgical intensive care units (ICUs) such as pneumonia in ventilated patients (VAPs), bloodstream infections (BSIs) and wound and urinary tract infections (UTIs). Healthcare-associated infections are becoming daunting for clinicians to manage due to the overwhelming increase in MDR isolates, which has put the practice and future of modern medicine at risk [1].

MDR bacteria of the utmost importance which frequently cause treatment failures are extended-spectrum β-lactamase (ESBL) and/or plasmid-mediated AmpC β-lactamase (p-AmpC) positive Enterobacterales, carbapenem-resistant Enterobacterales (CRE), carbapenem-resistant *Acinetobacter baumannii* (CRAB), carbapenem-resistant *Pseudomonas aeruginosa* (CRPA), methicillin-resistant *Staphylococcus aureus* (MRSA) and vancomycin-resistant *Enterococcus* spp. (VRE). They belong to the group of so-called ESKAPE pathogens [2].

ESBLs are plasmid-mediated β-lactamases which hydrolyse penicillin, expanded-spectrum cephalosporins (ESC) and monobactams. Antimicrobial therapy for infections caused by ESBL-producing Enterobacterales presents an additional challenge because the resistance genes for other antibiotic classes often reside on the same ESBL-encoding plasmids, thus limiting therapeutic options [3]. In the early 1980s, the major problems posed by ESBL-producing organisms were related to nosocomial infections caused by *K. pneumoniae* which produced TEM and SHV types of ESBLs [3]. First detected in 1986, CTX-M is a rising ESBL family, including more than 100 enzymes classified into five phylogenetic clusters [4]. Rather than arising by mutation, they were derived from the chromosomal β-lactamase genes of the *Kluyvera* spp. [4,5]. The rapid spread of CTX-M enzymes is associated with mobile genetic elements and particularly insertion sequences [5].

Resistance to ESC in *Enterobacter* spp., *Citrobacter* spp., *Morganella* morganii, *Providencia* spp. and *Serratia* spp. may develop through the expression of chromosomally encoded class C β-lactamases, also known as AmpC β-lactamases. The transfer of ampC genes to plasmids has resulted in their dissemination among Enterobacterales without the chromosomal ampC gene, with the consequence that ampC-encoded β-lactamases are now present in the species without chromosomal ampC genes such as *K. pneumoniae*, *P. mirabilis* and *Salmonella* spp. They are named plasmid-mediated AmpC beta-lactamases (p-AmpC) [6]. The above-mentioned β-lactamases confer resistance to monobactams, β-lactam/β-lactamase inhibitor combinations and to the first three generations of cephalosporins, but have no activity on cefepime or carbapenems [6].

Carbapenems are the antibiotics of choice for the treatment of infections due to ESBL- or AmpC-producing Enterobacterales; however, the rates of carbapenem resistance in Gram-negative bacteria are increasing. The mechanisms of resistance include the production of carbapenemases belonging to class A (KPC), B (IMP, VIM, NDM) or D (OXA-48, OXA-181), decreased permeability and efflux pump overexpression [7,8]. OXA-48, first described in *K. pneumoniae* in Turkey, is now the most widespread carbapenemase in this species in the majority of European countries, including Croatia [8]. The enzyme is capable of hydrolysing penicillin and carbapenems, but possesses poor activity against broad-spectrum cephalosporins. Multidrug resistance in OXA-48-producing organisms often results from the coproduction of ESBLs or p-AmpC β-lactamases [8].

Resistance genes in Enterobacterales are often carried on conjugative plasmids. A formal scheme of plasmid classification is based on incompatibility (Inc) groups: plasmids with the same replication control are incompatible and cannot reside in the same cell line, whereas plasmids with different replication controls are compatible and can be propagated in the same cell [9]. Classification is based on *rep* and *par* genes which are implicated in the replication, transfer and partitioning of the plasmids [9].

The production of carbapenemases, particularly KPC and NDM, is sometimes associated with hypervirulence in *K. pneumoniae* and severe infections in healthy patients [10].

*A. baumannii* is an important opportunistic pathogen that is rapidly evolving towards multidrug resistance. It causes serious infections in immunocompromised and debilitated patients, including wound infections, pneumonia in VAP, UTI, meningitis and BSI, associated with increased mortality [11]. CRAB has three important mechanisms that can be used to combat carbapenems, which include the production of carbapenemases, altered function porins and hyperexpression of drug efflux pumps [12]. CRAB produces carbapenemases belonging to molecular class A (GES, KPC), class B (VIM, SIM, IMP or NDM family) or class D (OXA enzymes) designated as carbapenem-hydrolysing class D oxacillinases (CHDL) [13].

Carbapenem-resistance in CRPA is most frequently attributed to the production of metallo-β-lactamases of the VIM, NDM and IMP families, although KPC and CHDL have also been reported. The alteration of OprD porins and the hyperexpression of the MEX efflux system contribute to resistance [14].

The difficulty in treating severe infections caused by CRE, CRPA and CRAB has led to the return of the use of colistin worldwide as the last option. Unfortunately, with the increasing use of polymyxins, colistin-resistant Gram-negative bacteria have been reported [15]. Polymyxin resistance is associated with specific modifications to the lipid A component of the bacterial lipopolysaccharide, resulting in a reduction in the negative charge of the outer membrane [16].

Methicillin resistance in staphylococci is mediated by the *mecA*/*mecC* gene-encoded altered PBP2a protein which is unable to bind to β-lactam antibiotics, except ceftobiprole and ceftaroline [17]. The mecA/mecC gene is located on a genetic element of the so-called *SCCmec* cassette. This element can also carry further resistance to other classes of antibiotics, so many MRSA strains, especially those circulating in hospitals, are still resistant to many other relevant antibiotics, which makes their treatment even more difficult.

Vancomycin-resistant enterococci (VRE) develop resistance to glicopeptydes through the substitution of D-alanil-D alanin with D-alanil-D lactat [18].

Resistance patterns vary between different countries and between hospitals in the same geographic region.

Here, we evaluated the MDR bacteria in surgical ICU in University Hospital Centre Zagreb (UHCZ) in order to determine their resistance phenotypes and the distribution of resistance genes. The study aimed to characterize β-lactamases and other resistance traits in Gram-negative bacteria isolated in surgical ICUs.

## 2. Materials and Methods

### 2.1. Bacterial Isolates

The study population comprised patients hospitalized in four surgical ICUs in UHCZ (surgery, cardiosurgery, neurosurgery and urology ICUs).

UHCZ is a 1724-bed university hospital and the largest medical centre in Croatia with all medical specialities including an organ and tissue transplantation department. It serves part of the Zagreb population and acts as a referral hospital for the whole Croatian population for specific patient/procedure groups, thus covering a population of about 4,000,000 people. Non-copy bacterial isolates (one per patient) were recovered from various clinical samples including clinically relevant (blood cultures, wound swabs, urine samples) and surveillance cultures (rectal swabs, stool, throat swab, etc.) from 1 October 2022 until 1 August 2023. The identification of isolates was carried out using an MALDI-TOF MS (matrix-assisted laser desorption ionization–time of flight mass spectrometry) Biotyper (Bruker, Daltonik GmbH, Bremen, Germany). For the measurement, MALDI Biotyper α-cyano-4-hdroxycinnamic acid (HCCA) was used as the MALDI matrix. A fresh solution was used every day. We added475 µL of ultrapure water, 500 µL of acetonitrile solution and 25 µL of 1005 tri-fluor-acetic acid to an Eppendorf tube to reach a final volume of 1 mL. The sample was vortexed. The content of the tube was called the standard solvent (SS). The inoculum size ranged from 10^4^ to 10^5^ CFU. The sample was applied to the metal tray and left to dry at room temperature, and then 1 µL of MALDI-TOF matrix was added [19]. When the sample was dry, the tray was inserted in the MALDI-TOF mass spectrometer and the was measurement started. The result was recorded.

As a part of routine diagnostics, all bacterial isolates were firstly tested using the Kirby–Bauer disk-diffusion test in line with EUCAST guidelines [20]. The isolate was considered to be multidrug resistant (MDR) if it was non-susceptible to at least one agent in three or more antimicrobial categories, and extensively drug resistant (XDR) if it was non-susceptible to at least one agent in all but two or fewer antimicrobial categories, while pandrug-resistance (PDR) was defined as non-susceptibility to all agents in all antimicrobial categories [21]. Isolates exhibiting an MDR or XDR phenotype were stored at −80 °C for further phenotypic and molecular analysis.

### 2.2. Antimicrobial Susceptibility Testing

The antimicrobial susceptibility of Gram-negative bacteria resistant to antibiotics listed in Table 1 was carried out by the broth microdilution method, using twofold serial dilutions referring to the Clinical Laboratory Standard Institute’s (CLSI) guidelines [22]. Breakpoints for colistin were defined by EUCAST, otherwise we applied those defined by the CLSI. *E. coli* ATCC25922 and *K. pneumoniae* ATCC 700603 were used as quality control strains. The species-specific panel of antibiotics is shown in Table 1. The susceptibility to ertapenem and ceftazidime-avibactam was tested only by the disk-diffusion method. Routine disk-diffusion was used to evaluate the Gram-positive bacteria’s susceptibility to antibiotics. MICs were determined only for vancomycin and teicoplanin.

### 2.3. Phenotypic Detection of β-Lactamases

ESBLs production was suspected in Enterobacterales based on reduced inhibition zones around cephalosporin disks and confirmed by a double disk synergy test (DDST) [23] and combined disk test using ESC and cefepime disks with and without clavulanic acid [22]. A difference of ≥5 mm between the zone diameters of either of the cephalosporin disks and their respective cephalosporin clavulanate disk is taken to be phenotypic confirmation of ESBL production. A double-disk synergy test with a disk supplemented with 500 µg cloxacillin placed between disks containing ceftazidime and cefotaxime on a lawn of the *K. pneumoniae*, *E. coli* and *P. mirabilis* isolates with reduced susceptibility to cefoxitin was performed in order to detect p-Amp-C. Distortion of the inhibition zones around ESC disks towards the central disk with cloxacillin was considered to be a positive result [6]. P-AmpC were confirmed by AmpC disk test according to Black [24]. A blank paper disk was impregnated with 20 µL Tris-EDTA. Three to five colonies of the test organism were applied to the surface of the disk. The disk was placed on the surface of MH agar previously inoculated with cefoxitin susceptible *E. coli* ATCC 25922, near to a cefoxitin disk. Distortion of the inhibition zone around the cefoxitin disk indicated enzymatic inactivation of cefoxitin.

All Enterobacterales with reduced susceptibility to carbapenems in the disk-diffusion test were subjected to immunochromatographic OKNV (OXA-48, KPC, NDM, VIM) testing for the purpose of routine diagnostics [25]. The production of carbapenemases was confirmed using the modified Hodge test (MHT) with an ertapenem disk as described previously [26]. In order to confirm or exclude KPC, MBLs or simultaneous production of KPC and MBL, the isolates were tested by combined disk tests with imipenem and meropenem alone and combined with PBA, 0.1 M EDTA or both [27]. To confirm carbapenem hydrolysis in carbapenem-resistant isolates, a CIM (carbapenem inactivation method) test was performed [28]. Overnight culture of the carbapenem-resistant test strain was suspended in saline and an ertapenem disk (10 µg) was placed in the suspension which was incubated for 2 h at 37 °C. As an indicator strain, *E. coli* ATCC 25922 was inoculated on Mueller–Hinton (MH) agar plates. The disk was removed after 2 h and placed in the middle of the plate. Carbapenem hydrolysis was confirmed if there was no inhibition zone, the zone was smaller than 14 mm or if there were colonies within the inhibition zone.

Drug efflux-pump inhibition assay was carried out on CRAB isolates. In brief, meropenem susceptibility testing was performed using the standard broth dilution test. Any change in the MIC of meropenem either in the absence or presence of carbonyl-cyanide-m-chlorophenythydrazone (CCCP) (20 µg/mL) was noted. A fourfold or greater reduction in the MIC values after addition of CCCP was considered significant [29]. In order to determine the effect of AmpC overproduction on the susceptibility of CRAB to carbapenems MICs of meropenem were evaluated with the addition of 200 mg/L of cloxacillin to the medium. An affirmative result would show at least a twofold decrease in meropenem MIC in the presence of cloxacillin [30].

*P. aeruginosa* was subjected to MHT [26] and an inhibitor-based test with EDTA [26] as previously described for Enterobacterales.

### 2.4. Molecular Analysis of Resistance Genes

The detection of resistance genes was carried out according to the species and antimicrobial resistance phenotype. The bacterial cells were boiled in sterile water at 95 °C for 10 min and the supernatant was used as template DNA. The Enterobacterales were checked for the presence of acquired resistance genes by standard PCR amplification, including broad spectrum β-lactamase and ESBL genes (*bla*_SHV_, *bla*_TEM_, *bla*_CTX-M_) [31,32,33] and fluoroquinolone resistance determinants (*qnr*A, *qnr*B and *qnr*S) [34]. Colistin-resistant *K. pneumoniae* isolates were additionally checked for plasmid mediated colistin resistance genes such as *mcr*-1 and *mcr*-2 [35]. The five clonal lineages of CTX-M β-lactamases 1, 2, 8, 9 and 25 [36], p-AmpC [37] and carbapenemases-encoding genes, class A (*bla*_KPC_), class B or metallo-β-lactamases known as MBLs (*bla*_VIM_, *bla*_IMP_, and *bla*_NDM_) and class D carbapenemases or carbapenem-hydrolysing oxacillinases or CHDL (*bla*_OXA-48-like_) were verified with multiplex PCR [38]. The flanking regions of *bla*_CTX-M_ genes were analysed by combining the forward primer for IS*Ecp1* and IS*26* with the universal reverse primer for *bla*_CTX-M_ genes (MA 3) [39]. Specific primers for IS*1999* combined with forward and reverse primers for *bla*_OXA-48-like_ were used to determine the upstream or downstream position of the insertion sequence [40]. The positive control strains producing TEM-1, TEM-2 and SHV-1 and SHV-2 were kindly provided by Prof. Adolf Bauernfeind (Max von Pettenkofer Institute, Munich, Germany), CTX-M-15 by Prof. Neil Woodford (Health Protection Agency, London, UK), KPC-2 by Prof. Fred Tenover (Stanford University School of Medicine) and OXA-48 by Dr. Yvonne Pfeifer (Robert Koch Institute, Wernigerode, Germany).

In *A. baumannii* isolates, amplification of genes encoding KPC, MBLs (*bla*_VIM_, *bla*_IMP_, *bla*_SIM_ and *bla*_NDM_) and CHDL *(bla*_OXA-51-like_, *bla*_OXA-23-like_, *bla*_OXA-24/40-like_, *bla*_OXA-58-like_, and *bla*_OXA-143-like_) was performed by PCR [38,41]. The surrounding regions of *bla*_OXA-51_ and *bl*a_OXA-23_ genes were assessed by combining primers for IS*Aba1* with forward and reverse primers for *bla*_OXA-51_ and *bla*_OXA-23_ [42].

*P. aeruginosa* was subjected to PCR for acquired carbapenemase genes [38].

### 2.5. Genotyping by Inter-Array Kit CarbaResist

Four *K. pneumoniae* isolates were genotyped by an Inter-array chip according to the manufacturer’s recommendations (Inter-array fzmb GmbH, Bad Langensalza, Germany). The Inter-array genotyping Kit CarbaResist detects broad-spectrum β-lactamases, p-AmpC, ESBLs and carbapenemases and numerous other resistance genes (https://www.inter-array.com/further-genotyping-kits (accessed on 1 February 2024)). RNA-free, unfragmented genomic DNA was isolated from pure culture of the test strains and amplified and then internally labelled with biotin-dUDP using the linear PCR amplification protocol and only the antisense primer of the different targets. Single-stranded DNA (ssDNA) reaction products were obtained. The biotin-labelled ssDNA was transferred to the ArrayWell and hybridised to DNA oligonucleotide microarrays with 230 probes for different β-lactam, aminoglycoside, fluoroquinolone, sulphonamide, trimethoprim and colistin resistance genes. HRP-conjugated streptavidin was bound to the hybridised biotin-labelled ssDNA stains and visualised by enzymatic reaction. The INTER-VISION Reader was used to evaluate the spots and their intensities automatically on the basis of a digital image of the microarray. The samples obtained from the strains tested in the study were automatically analysed for the presence or absence of specific probes, cross-checked against a database and then information about existing resistances was output.

### 2.6. Conjugation

The overnight cultures of enterobacterial wild-type strains and *E. coli* J65 recipient strains resistant to sodium azide were suspended in Brain–Heart Infusion (BHI) broth and incubated overnight at 35 °C without shaking [43]. MacConkey agar supplemented with either ertapenem (0.5 mg/L), cefotaxime (2 mg/L) or cefoxitin (10 mg/L) to supress the growth of recipient strain and sodium azide (100 mg/L) to inhibit the donor strains, was used to select transconjugant clones harbouring resistance to β-lactam antibiotics and sodium azide. Colonies growing on combined plates were subjected to identification by MALDI-TOF, and if confirmed to be an *E. coli* transconjugant, were further subjected to antibiotic susceptibility testing. PCR was used to test for the *bla* genes in transconjugants, as described for clinical isolates.

### 2.7. Characterization of Plasmids

PCR-based replicon typing (PBRT) [9] was used for molecular typing of plasmids conferring resistance in Enterobacterales. Eighteen pairs of primers were used, including five multiplex and three simplex PCR tests in order to assess the plasmid incompatibility group. Since L/M plasmids which usually carry *bla*_OXA-48_ genes are difficult to detect with the original protocol, an updated protocol by Carattoli was applied [44].

Plasmid extractions obtained from transconjugant strains were subjected to PCR for the detection of carbapenemase and ESBL-encoding genes in order to determine the resistance gene content of the transconjugants. PBRT was also applied to transconjugants to identify incompatibility groups, such as in their respective donors. Positive control strains for PBRT were obtained from Dr A. Carattoli (Instituto Superiore di Sanita, Rome, Italy).

In *A. baumannii* plasmid incompatibility groups were analysed by PBRT according to the scheme developed for *A. baumannii* [45].

### 2.8. String Test

String test was attempted on *K. pneumoniae* isolates in order to determine hypervirulence, by stretching a mucoviscous string from the colony using a standard bacteriologic loop, as described previously [10]. If a viscous string >5 mm was formed, the isolate was defined as hypermucoviscous.

### 2.9. Genotyping of the Isolates

Clonal types of two representative *K. pneumoniae* isolates (29 and 30) were determined by MLST, in line with Institute Pasteur scheme (http://bigsdb.web.pasteur.fr/klebsiella/klebsiella.html (accessed on 1 February 2024)) [46].

Sequence groups (SGs 1–3) corresponding to international clonal lineages (ICL I–III) were analysed in all isolates by multiplex PCR in *A. baumannii* [47].

## 3. Results

### 3.1. Patients and Isolates

**Figure 1:**
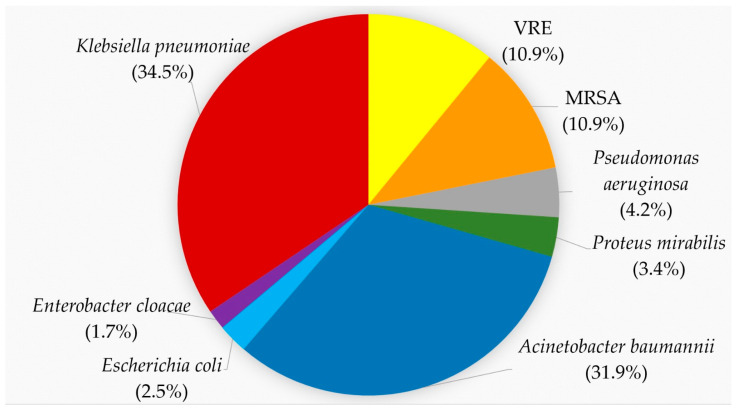
Proportion of MDR isolates according to the species. *K. pneumoniae*, red, 34.5%; *A. baumannii*, blue, 31.9%; VRE, yellow, 10.9%; MRSA, orange, 10.9%; *P. aeruginosa*, gray, 4.2%; *P. mirabilis*, green, 3.4%; *E. coli*, light blue, 2.5%; *E. cloacae*, purple, 1.7%.

In total, 119 MDR pathogens were recovered during the study period: forty-one *K. pneumoniae*, thirty-eight *A. baumannii*, five *P*. *aeruginosa*, four *P. mirabilis*, three *E. coli* and two *E. cloacae* complexes. Among the Gram-positive bacteria there were thirteen MRSA and VRE, respectively (Figure 1). The MALDI-TOF score for *K. pneumoniae* ranged from 2.02 to 2.37 and for *A. baumannii* from 2.17 to 2.32. The rwo *E. coli* had mass spectra scores of 2.43 and 2.45, respectively. Examples of these scores are given in Supplementary Table S1.

Out of the 119 isolates, 42 (35%) were recovered from clinically relevant specimens (blood cultures, BAL, wound swabs) and the rest were from surveillance cultures.

### 3.2. Laboratory Analysis of Bacterial Isolates

#### 3.2.1. Enterobacterales

##### *K. pneumoniae* 

All 41 *K. pneumoniae* isolates exhibited uniform resistance to amoxicillin-clavulanate (Table 1). The resistance rates for other antibiotics were as follows: 97.5% (n = 40) for ESC, 95.1% for ciprofloxacin (n = 39), 90.2% (n = 37) for ertapenem, 70.7% (n = 29) for cefepime, meropenem and piperacillin-tazobactam, 68.2% (n = 28) for imipenem and 58.5% (n = 24) for gentamicin, as shown in Table 1. Amikacin remained active against 70.7% of the isolates. Intermediate susceptibility to piperacillin-tazobactam (susceptibility with increased exposure), identified with the dilution method, was recorded for 29.3% (n = 12) of the isolates which were found to be resistant in the disk-diffusion test. Ceftazidime-avibactam and colistin remained active against the majority of isolates with only 4.8% (n = 2) and 7.3% (n = 3) of the isolates being resistant. If the EUCAST criteria were applied, which are less stringent, 63.4% (n = 26) of the isolates would be resistant to meropenem, but only 17.7% (n = 7) would be resistant to imipenem. Colistin and imipenem were the most efficient antibiotics with MIC_90_ of 32 µg/mL. The details of the antimicrobial susceptibility patterns are illustrated in Table 1. In terms of susceptibility to antibiotics, three strains were XDR and susceptible only to ceftazidime-avibactam, all of the others were MDR.

**Table 1:** Minimum inhibitory concentrations of various antibiotics and the rate of resistant *K. pneumoniae* isolates.

Antibiotic	MIC Range	MIC_50_	MIC_90_	Number and % of Resistant Strains
amoxycillin-clavulanate	>128–>128	>128	>128	41/41 (100.0%)
piperacillin-tazobactam	64–>128	128	>128	29/41 (70.7%)
cefuroxime	>128–>128	>128	>128	41/41 (100.0%)
ceftazidime	0.5–>128	128	>128	40/41 (97.5%)
cefotaxime	1–>128	>128	>128	40/41 (97.5%)
ceftriaxone	0.5–>128	128	>128	40/41 (97.5%)
Cefepime	0.25–>128	64	>128	29/41 (70.7%)
imipenem	0.06–64	8	32	28/41 (68.2%)
meropenem	0.06–64	16	64	29/41 (70.7%)
gentamicin	0.25–>128	16	>128	24/41 (58.5%)
amikacin	8–>128	16	128	12/41 (29.3%)
ciprofloxacin	0.25–>128	64	>128	39/41 (95.1%)
colistin	0.06–128	0.5	32	3/41 (7.3%)

**Figure 2:**
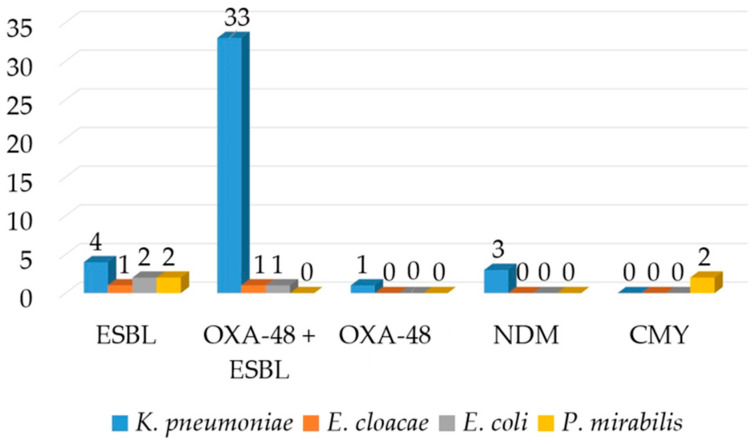
Distribution of various β-lactamases according to the species.

ESBLs were detected by DDST and confirmed by the combined disk test in 90.2% (n = 37) of the isolates. The OKNV test identified OXA-48 carbapenemase in 82.9% (n = 34) of the isolates and NDM in 7.3% (n = 3) of the isolates (Figure 2). PCR confirmed the OKNV results from the hospital routine laboratory. The OXA-48-producing organisms demonstrated a uniform resistance to ertapenem, but heterogeneous resistance to imipenem and meropenem with MICs ranging from 1 to 64 mg/L, and thus were classified as susceptible, intermediate susceptible (susceptible at increased exposure) and resistant according to CLSI. The MHT and CIM tests had a low sensitivity and detected only 60.9% (n = 25) and 73.1% (n = 30) of the isolates positive in OKNV and PCR. However, the inhibitor-based test with EDTA produced positive result with all three isolates being positive for NDM in OKNV. PCR detected *bla*_CTX-M_ genes which were proved to belong to group 1 by a multiplex PCR in all of the isolates that were phenotypically positive for ESBL and *bla*_TEM_ genes in eight isolates. The *bla*_TEM_ genes generated a TEM-1 allelic variant. The *bla*_CTX-M_ genes in 25 isolates were linked to an upstream IS*Ecp1*-like element. IS*1999* was detected upstream of the *bla*_OXA-48_ genes in 12 OXA-48-like positive isolates. PCR for *mcr* genes gave a negative result for three of the colistin-resistant isolates. Twelve OXA-48 isolates conjugated successfully at the frequency of 10^−5^ to 10^−6^ (transconjugant/donor), in contrast to NDM, but the other resistance determinants were not co-transferred. The PCR also showed that no ESBL-resistance determinants had co-transferred alongside carbapenem resistance. Attempts to transfer cefotaxime resistance were not successful. The transconjugants exhibited lower carbapenem MICs compared to the wild-type isolates. PBRT showed that the *bla*_NDM_ genes were associated with the IncX plasmid in all three isolates, and *bla*_OXA-48-like_ with IncL genes in approximately half of the strains (n = 18). The IncW plasmid was detected in two isolates positive for OXA-48 and CTX-M. Hypervirulence was detected in one isolate positive for OXA-48-like carbapenemase.

The inter-array chip test identified a wide variety of β-lactam resistance genes including *bla*_OXA-48_, *bla*_CTX-M-15_, *bla*_SHV_ and *bla*_OXA-1_. Among the aminoglycoside resistance genes, *aac(6”)-Ib* was present in all four while only one isolate tested positive for the *grm* and *arm* genes that encode panaminoglycoside resistance, respectively (Table 2). The *dfrA14* genes for trimethoprim resistance were harboured by all four isolates with one being positive for *dfrA5* as well (Table 2). All of the tested isolates were positive for the efflux pump genes *oqxA and oqxB*. Two isolates (29 and 30) were found to belong to ST101 and ST5879, respectively.

##### *E. cloacae* Complex

Both of the *E. cloacae* complex isolates exhibited resistance to ESC, gentamicin and ciprofloxacin and susceptibility to imipenem, meropenem and colistin, but showed variable susceptibilities to piperacillin-tazobactam, cefepime and ertapenem. Both isolates tested positive for ESBLs, whereas the Hodge and CIM test yielded a positive result in one of the tested isolates in which OKNV and PCR identified OXA-48. PCR identified CTX-M cluster one and OXA-48 encoding genes (Figure 2). Similarly, as with *K. pneumoniae*, only ertapenem resistance was transferable to the *E. coli* recipient strain.

##### *E. coli* 

All three *E. coli* isolates expressed resistance to amoxicillin, cefotaxime and ceftriaxone. Resistance to ertapenem and imipenem was recorded in one isolate. Ciprofloxacin resistance was displayed by two and gentamicin resistance by one isolate. Phenotypic testing confirmed ESBL in all isolates, while the Hodge and CIM test yielded a positive result in one isolate with reduced susceptibility to carbapenems. All three isolates harboured *bla*_CTX-M_ genes with one being positive for *bla*_OXA-48-like_ as well.

##### *P. mirabilis* 

Two *P. mirabilis* isolates displayed the ESBL and AmpC phenotypes, respectively, which were confirmed by inhibitor-based tests and the latter with a AmpC disk as well. All were resistant to co-amoxiclav, ESC, gentamicin and ciprofloxacin. Piperacillin-tazobactam and cefepime remained susceptible. ESBL-producing organisms expressed *bla*_CTX-M-9_ genes, whereas ampC-producing organisms possessed *bla*_CMY_ genes, as illustrated in Figure 2. The *bla*_CMY_ gene was preceded by the IS*Ecp* element upstream of the gene.

The conjugation experiments failed to transfer cefotaxime, cefoxitin and ertapenem resistance to the *E. coli* recipient strain.

**Table 2:** Analysis of antibiotic resistance genes by inter-array chip method.

Isolate	β-Lactam	Aminoglycosides	Trimethoprim	Efflux Pump
*K. pneumoniae*	*bla*_OXA-48_	*aac/6”)-Ib*	*dfrA5*	*oqxA*
29	IS*Ecpbla*_CTX-M-15_	*grm*	*dfrA14*	*oqxB*
41968	*bla*_SHV_			
	*bla*_oxa-1_			
*K. pneumoniae*	*bla*_OXA-48_	*aac/6”)-Ib*	*dfrA14*	*oqxA*
30	IS*Ecpbla*_CTX-M-15_			*oqxB*
41968	*bla*_SHV_			
	*bla*_oxa-1_			
*K. pneumoniae*	*bla*_OXA-48_	*aac/6”)-Ib*	*dfrA14*	*oqxA*
31	IS*Ecpbla*_CTX-M-15_			*oqxB*
82113	*bla*_SHV_			
	*bla*_oxa-1_			
*K. pneumoniae*	*bla*_OXA-48_	*aac/6”)-Ib*	*dfrA14*	*oqxA*
33	IS*Ecpbla*_CTX-M-15_	*arm*		*oqxB*
117640	*bla*_SHV_			
	*bla*_oxa-1_			

#### 3.2.2. *A. baumannii* 

Uniform resistance to cefepime, ceftazidime, meropenem, imipenem, ciprofloxacin and gentamicin was observed (Table 3). A high resistance rate was observed for piperacillin-tazobactam (86.8%, n = 33), whereas ampicillin-sulbactam remained active against 68.4% (n = 26) of the isolates (Table 2). No resistance to colistin was observed. In terms of susceptibility to antibiotics, all of the isolates were XDR.

The MICs of imipenem and meropenem were not reduced by either the presence of the efflux inhibitor or cloxacillin. The Hodge and CIM tests exhibited a low sensitivity with only 11 (28.8%) and 10 (26.3%) of the CRAB strains being positive, respectively. All of the isolates tested positive in the inhibitor-based test with EDTA indicating the production of an MBL, but the PCR for common class B carbapenemases yielded a negative result. PCR identified *bla*_OXA-23-like_ genes in all isolates which harboured a plasmid belonging to the Inc group 2-encoding aci2-replicase gene. All except two isolates were asigned to SG1 corresponding to IC2.

**Table 3:** Antibiotic susceptibility of *A. baumannii* isolates.

Antibiotic	MIC Range	MIC_50_	MIC_90_	Number and % of Resistant Strains
piperacillin-tazobactam	32–>128	>128	>128	33/38 (86.8%)
ampicillin-sulbactam	1–128	8	128	12/38 (31.5%)
ceftazidime	128–>128	>128	>128	38/38 (100.0%)
cefepime	32–>128	>128	>128	38/38 (100.0%)
imipenem	32–>128	128	>128	38/38 (100.0%)
meropenem	64–>128	>128	>128	38/38 (100.0%)
gentamicin	32–>128	128	>128	38/38 (100.0%)
amikacin	32–>128	64	>128	27/38 (71.1%)
ciprofloxacin	8–>128	128	>128	38/38 (100.0%)
colistin	1–2	1	1	0/38 (0.0%)

#### 3.2.3. *P. aeruginosa* 

Uniform resistance to all of the β-lactam antibiotics was recorded in *P. aeruginosa* isolates except for ceftolozane/tazobactam which showed activity against two isolates. Amikacin and colistin were efficient against all of the isolates. The modified Hodge test and inhibitor-based EDTA test yielded positive results for three of the isolates, being indicative of an MBL. PCR confirmed the genes encoding VIM-MBLs in three of the isolates that were phenotypically positive for an MBL.

#### 3.2.4. Gram-Positive Bacteria

The majority of the tested antibiotics were highly active against MRSA. No resistance to sulfamethoxazole-trimethoprim, vancomycin, teicoplanin or linezolid was recorded. Only one isolate showed intermediate susceptibility to ciprofloxacin while all other were resistant. Only one isolate tested showed resistance to gentamicin. VRE isolates were uniformly resistant to ampicillin and ciprofloxacin, but all were susceptible to linezolid and daptomycin. In contrast to MRSA, gentamicin exhibited poor activity with only two isolates (15.4%) being susceptible. All strains exhibited a VAN-A phenotype with high MIC values for both vancomycin and teicoplanin.

## 4. Discussion

There are several studies on multidrug-resistant bacteria in surgical ICUs, but a molecular analysis of resistance genes is missing from the majority of publications, which would enable comparisons with other studies [48,49].

We found that OXA-48-producing *K. pneumoniae* and CRAB were the dominant causative agents of infections in the surgical ICUs. All except one OXA-48 and two NDM-1-positive *K. pneumoniae* harboured additional ESBL that conferred or increased resistance to ESC. The Hodge and CIM tests failed to identify many of the carbapenemase-producing Enterobacterales. The higher carbapenem MICs observed for the clinical isolates than those for the respective transconjugants may likely be attributable to porin loss or the hyperexpression of efflux pumps. Cumulative MIC values and the rate of resistant isolates were higher for meropenem, in spite of the fact that oxacillinases preferentially hydrolyse imipenem. This could be attributed to additional resistance mechanisms such as hyperexpression of efflux systems which affect meropenem more than imipenem.

*K. pneumoniae* was the dominant host of OXA-48, but the enzyme has also been reported in *E. coli* and *E. cloacae*. Analysis of the genetic context of *bla*_OXA48-like_ genes identified the IS*1999* element which functions as a promoter and increases the expression of the gene [50]. IS*Ecp* detected upstream of the *bla*_CTX-M_ genes in some of the isolates is responsible for the mobilization event and increases the level of resistance [51]. The studies carried out in Africa also found CRKP and CRAB to be the most frequent MDR pathogens in ICUs [52]. In the African study, no molecular analysis of resistance genes was carried out. Other European studies found OXA-48 to be dominant in Balkan countries such as Bulgaria, Serbia and Slovenia which are neighbouring to Croatia, but KPC and NDM were reported as well [53]. However, in our study KPC was not detected. In Italy, OXA-48 was replaced recently by a new variant, OXA-181. In the Italian study, the *bla*_OXA-181_ genes were also carried by the L plasmid similarly to *bla*_OXA-48_ in the present investigation [54]. PCRs for plasmid-mediated *mcr* genes gave negative for in all three colistin resistant strains and therefore the resistance can probably be attributed to the disruption of the *mgr*B gene as described previously in Italy [55]. ST101, reported in this study in the OXA-48 producing isolate, was previously identified in wastewater *K. pneumoniae* isolates in Finland [56] and in Italy [57], and in both cases was linked to KPC positivity. OXA-48 was carried by the L type plasmid, reinforcing the hypothesis that the current spread of OXA-48-carrying genes in different species (*K. pneumoniae*, *E. cloacae* and *E. coli*) is mainly the consequence of the diffusion of an epidemic, conjugative plasmid.

Previous studies have shown that plasmids carrying *bla*_OXA48_ genes, share similar features, in that they have an incL/M backbone and are very similar in size (60–70 kb), suggesting their wide dissemination in different species and countries via an epidemic plasmid [8]. *E. cloacae* complex, *P. mirabilis* and *E. coli* occurred only sporadically with only a few isolates being analysed in the present study.

Interestingly, ESBLs found in P. *mirabilis* belonged to CTX-M group 9 in contrast to other European studies which identified TEM-92 [58]. Amp β-lactamase found in the *P. mirabilis* isolates turned out to be CMY-16, the allelic variant previously reported in Italy [59]. It is the most widespread allelic variant in Europe, particularly in southern regions [60]. The CMY-producing organisms exhibited resistance to ESC but susceptibility to cefepime and piperacillin-tazobactam. The IS*Ecp*-1 like element linked to *bla*_CMY_ and *bla*_CTX-M_ genes was previously shown to play a key role in mobilization, but also in chromosomal incorporation of the genes [60]. This could explain the nontrasferability of cefoxitin resistance in the conjugation experiments in our study. In African countries, carbapenemases were detected among *P. mirabilis* isolates but the types of enzymes were not determined [61]. This is in contrast with our investigation in which all isolates tested were susceptible to carbapenems.

The *E. cloacae complex* was very rare among the tested isolates and OXA-48 was the only carbapenemase detected in this species. This is in contrast with the previous reports from 2014 which identified VIM-1 and NDM-1 as the only carbapenem-resistance trait among *Enterobacte*r spp. in Croatia [62,63,64]. Similarly, as in previous studies carbapenemase-encoding genes were accompanied by ESBL-encoding genes, such as *bla*_CTX-M-15_. In the Far East, IMP variants are dominant in the *E. cloacae* complex [65]. In African countries, the OXA-48, OXA-181 and NDM variants were found as carbapenem resistance determinants in *E. cloacae* complex isolates [66]. In the majority of reports, carbapenemases were accompanied by other antibiotic resistance determinants showing the amazing capacity of this species to accumulate resistance genes.

OXA-23 was the most prevalent CHDL in *A. baumannii.* Both the CIM and Hodge tests missed the majority of OXA-23-producing isolates. This could be explained by the low hydrolysis of carbapenems by CHDL. The high MICs of carbapenems are probably attributed to combined mechanisms such as porin loss or efflux. The inhibitor-based test with EDTA yielded a false positive result in all *A. baumannii* isolates. The phenomenon was reported before and is due to the fact that oxacillinases convert to a less active form in the presence of the metal chelator leading to the enlargement of the inhibition zone in the presence of EDTA [67]. The drug efflux inhibition assay and cloxacillin assay failed to reduce the meropenem MICs, and thus no changes in or restorations of susceptibilities were recorded, suggesting that neither the hyperexpression of efflux pumps nor chromosomal AmpC β-lactamase affected the carbapenem susceptibility in *A. baumannii.* Other European studies corroborated the rapid diffusion of OXA-23 in *A. baumann*ii [68] although OXA-24/40-like and OXA-58-like genes were identified in the minority of the isolates. MBLs were found in 2% of the isolates in the German study [69]. This is in contrast with our study which found MBLs only in *K. pneumonia* and *P. aeruginosa*. According to the national surveillance studies published at https://iskra.bfm.hr/wp-content/uploads/2022/11/Knjiga-2021-za-web-final.pdf (accessed on 1 February 2024), *A. baumannii* is one of the most resistant pathogens with over 85% of isolates being carbapenem-resistant. However, MBLs were not found so far.

This pathogen is characterized by an intrinsic resistance to a number of drugs, as well as an outstanding ability to acquire the determinants of antibiotic resistance through horizontal gene transfers leading to high level resistance to almost all available antibiotics. Uniform in vitro susceptibility was retained only for colistin.

*P. aeruginosa* harboured MBLs belonging to the VIM family with some of the CRPA strains being negative for carbapenemases and the reduced carbapenem susceptibility is probably owed to porin loss or combined mechanisms. VIM-2 was previously reported in the surgical ICUs of UHCZ a long time ago [70] and is believed to be endemic in the hospital, probably colonizing inanimate objects and surfaces which serve as a source of patients’ colonization. Other European studies found VIM-2, IMP-1 and NDM-1 to confer carbapenem resistance in *P. aeruginosa* [71].

Ceftazidime/avibactam and colistin exhibited the best activity against *K. pneumoniae* isolates with less than 10% of the isolates showing resistance. Resistance to ceftazidime/avibactam was related to the production of MBLs. The implementation of the immunochromatographic OKNV test in routine diagnostic testing for rapid identification of the carbapenemase class enables clinical microbiologist to provide advice about therapy before susceptibility testing is finished. Ceftazidime-avibactam is recommended if OXA-48 is confirmed while novel inhibitor combinations are ruled out if MBL is identified.

The strength of this manuscript is the detailed molecular analysis of resistance determinants, whereas the weakness is the fact that all of the isolates originated from the same hospital and hospital ward. Moreover, new molecular methods such as whole-genome sequencing were not applied to enable analysis of the whole resistome of the isolates. Gram-positive organisms were not subjected to molecular analysis of resistance genes. Hypervirulence was detected only by the phenotypic method without carrying out PCRs for the four virulence genes.

## 5. Conclusions

OXA-48, ESBLs and p-AmpC were the dominant resistance traits among our isolates The study underscores the urgent need for surveillance and intervention strategies in order to curb the escalating rates of carbapenem resistance in Enterobacterales and *A. baumannii*. The findings of this study provide valuable insight into the resistance landscape, informing clinical approaches and guiding future research targeting this critical threat to global health.

## Data Availability

The data presented in the study are available on request from the corresponding author.

## Supplementary Materials

The following supporting information can be downloaded at: https://www.mdpi.com/article/10.3390/pathogens13050411/s1, Table S1: Mass spectra of representative strains.
