# Supplementary material for: Multidrug-Resistant Bacteria in Surgical Intensive Care Units: Antibiotic Susceptibility and β-Lactamase Characterization
Source: Pathogens. 2024 May 15;13(5):411. doi: 10.3390/pathogens13050411 (PMC11124292; doi:10.3390/pathogens13050411)
Supplement: Supplementary file 1 [file pathogens-13-00411-s001.zip › pathogens-2977339-supplementary.pdf]

*Result overview table--continued from previous page*

| Sample Name | Sample ID | Organism (best match) | Score Value | Organism (second-best match) | Score Value |
|---|---|---|---|---|---|
| D4 (+++) (A) | 670 (standard) | Staphylococcus epidermidis | 2.22 | Staphylococcus epidermidis | 2.22 |
| D5 (+++) (A) | 36 (standard) | Escherichia coli | 2.43 | Escherichia coli | 2.24 |
| D6 (+++) (A) | 36 OKO MEM (standard) | Enterococcus faecium | 2.49 | Enterococcus faecium | 2.40 |
| D7 (+++) (A) | 37 (standard) | Escherichia coli | 2.45 | Escherichia coli | 2.24 |
| D8 (+++) (A) | 37 OKO MEM (standard) | Enterococcus faecium | 2.40 | Enterococcus faecium | 2.32 |
| D9 (+++) (A) | 128 1 (standard) | Acinetobacter baumannii | 2.32 | Acinetobacter baumannii | 2.32 |
| D10 (+++) (A) | 2 (standard) | Staphylococcus epidermidis | 2.20 | Staphylococcus epidermidis | 2.12 |
| D11 (+++) (A) | MC (standard) | Acinetobacter baumannii | 2.34 | Acinetobacter baumannii | 2.29 |
| D12 (+++) (A) | 145 MC OKR (standard) | Providencia stuartii | 2.24 | Providencia stuartii | 2.22 |
| E1 (+++) (A) | MC RAZL (standard) | Providencia stuartii | 2.54 | Providencia stuartii | 2.50 |
| E2 (+++) (A) | CNA BIJ (standard) | Providencia stuartii | 2.41 | Providencia stuartii | 2.37 |
| E3 (+++) (A) | CNA SIV (standard) | Providencia stuartii | 2.50 | Providencia stuartii | 2.46 |
| E4 (+++) (A) | 147 MC (standard) | Acinetobacter baumannii | 2.36 | Acinetobacter baumannii | 2.17 |
| E5 (+++) (A) | CNA KR (standard) | Staphylococcus aureus | 2.56 | Staphylococcus aureus | 2.50 |
| E6 (+++) (A) | CNA SIT (standard) | Corynebacterium striatum | 2.40 | Corynebacterium striatum | 2.36 |
| E7 (+++) (A) | 672 (standard) | Raoultella ornithinolytica | 2.22 | Raoultella ornithinolytica | 2.01 |

*Result overview table--continued on next page*

| Result overview table--continued from previous page | | | | | |
|---|---|---|---|---|---|
| Sample Name | Sample ID | Organism (best match) | Score Value | Organism (second-best match) | Score Value |
| F12 (+++) (C) | 3-1 (standard) | Klebsiella oxytoca | 2.21 | Klebsiella oxytoca | 2.12 |
| G1 (+++) (A) | 4917-1-1 (standard) | Micrococcus luteus | 2.03 | Micrococcus luteus | 1.94 |
| G2 (-) (C) | X-1 (standard) | **no peaks found** | 0.00 | **no peaks found** | 0.00 |
| G3 (+++) (A) | 4664-1-1 (standard) | Staphylococcus haemolyticus | 2.18 | Staphylococcus borealis | 1.97 |
| G4 (+++) (C) | 2-1 (standard) | Staphylococcus epidermidis | 2.35 | Staphylococcus epidermidis | 2.24 |
| G5 (+++) (C) | 3-1 (standard) | Staphylococcus epidermidis | 2.22 | Staphylococcus epidermidis | 2.00 |
| G6 (+++) (A) | KP 51-1 (standard) | Klebsiella pneumoniae | 2.24 | Klebsiella pneumoniae | 2.23 |
| G7 (+++) (A) | KP 52-1 (standard) | Klebsiella pneumoniae | 2.12 | Klebsiella pneumoniae | 2.02 |
| G8 (+++) (A) | 4763-1-1 (standard) | Citrobacter freundii | 2.30 | Citrobacter freundii | 2.27 |
| G9 (+++) (C) | 2-1 (standard) | Klebsiella pneumoniae | 2.38 | Klebsiella pneumoniae | 2.37 |
| G10 (+++) (C) | 3-1 (standard) | Enterococcus faecalis | 2.39 | Enterococcus faecalis | 2.35 |
| G11 (+++) (C) | 4-1 (standard) | Enterococcus faecium | 2.52 | Enterococcus faecium | 2.48 |
